# Binding Site Prediction for Protein-Protein Interactions and Novel Motif Discovery using Re-occurring Polypeptide Sequences

**DOI:** 10.1186/1471-2105-12-225

**Published:** 2011-06-02

**Authors:** Adam Amos-Binks, Catalin Patulea, Sylvain Pitre, Andrew Schoenrock, Yuan Gui, James R Green, Ashkan Golshani, Frank Dehne

**Affiliations:** 1 School of Computer Science, Carleton University, Ottawa, ON K1S5B6, Canada; 2 Department of Biology, Carleton University, Ottawa, ON K1S5B6, Canada; 3 Department of Systems and Computer Engineering, Carleton University, Ottawa, ON K1S5B6, Canada

## Abstract

**Background:**

While there are many methods for predicting protein-protein interaction, very few can determine the specific site of interaction on each protein. Characterization of the specific sequence regions mediating interaction (binding sites) is crucial for an understanding of cellular pathways. Experimental methods often report false binding sites due to experimental limitations, while computational methods tend to require data which is not available at the proteome-scale. Here we present PIPE-Sites, a novel method of protein specific binding site prediction based on pairs of re-occurring polypeptide sequences, which have been previously shown to accurately predict protein-protein interactions. PIPE-Sites operates at high specificity and requires only the sequences of query proteins and a database of known binary interactions with no binding site data, making it applicable to binding site prediction at the proteome-scale.

**Results:**

PIPE-Sites was evaluated using a dataset of 265 yeast and 423 human interacting proteins pairs with experimentally-determined binding sites. We found that PIPE-Sites predictions were closer to the confirmed binding site than those of two existing binding site prediction methods based on domain-domain interactions, when applied to the same dataset. Finally, we applied PIPE-Sites to two datasets of 2347 yeast and 14,438 human novel interacting protein pairs predicted to interact with high confidence. An analysis of the predicted interaction sites revealed a number of protein subsequences which are highly re-occurring in binding sites and which may represent novel binding motifs.

**Conclusions:**

PIPE-Sites is an accurate method for predicting protein binding sites and is applicable to the proteome-scale. Thus, PIPE-Sites could be useful for exhaustive analysis of protein binding patterns in whole proteomes as well as discovery of novel binding motifs. PIPE-Sites is available online at http://pipe-sites.cgmlab.org/.

## Background

In recent years, large-scale protein sequencing has generated a tremendous amount of data at the proteome level. However, biomedical significance of these results relies on the determination of protein function at this scale. Protein-protein interactions (PPIs) are the main mechanism behind biological pathways [1] and as such are at the core of a functional understanding of the cell under normal and pathological conditions. Characterization of the sequence regions that give rise to protein binding sites responsible for interaction (interaction or binding site) opens the door to modulation of these pathways and is crucial to targeted drug design.

Large-scale protein-protein interaction (PPI) studies are bottlenecked by existing experimental and computational methods. Experimental assays such as yeast two-hybrid (Y2H) [2,3] or TAP-tagging [4,5] have been adapted to the proteome scale but lag in terms of throughput and robustness. For example, Y2H underreports interactions involving membrane and cytosol proteins because the assay requires the proteins to interact with the nucleus [6]. Deletion experiments, for binding site determination, are even more time-consuming and often produce very coarse data unsuitable for detailed study of binding sites. In addition, deletion experiments may create false positives because the deletion of assumed binding sites may alter the conformation of the protein and thereby disable the protein interaction without directly mediating binding of the two proteins.

Computational tools promise significant advantages in response time and cost-effectiveness. However, molecular modelling approaches such as protein docking [7,8] require 3D structure data not available at the proteome scale and are computationally prohibitive at that scale. 3D structure prediction from primary structure is also infeasible at this scale, requiring world-wide distributed computing efforts for several months to process a single protein [9].

Direct sequence-based methods are capable of overcoming some of these limitations by performing binding site prediction from domain motifs [10-13] or directly from a machine learning model [14-17]. However, in each case, model training is limited by available data since consensus motifs must be manually curated, and training sets in the form of high-quality binding site location databases are scarce and costly to produce. Additionally, methods based on domain-motif binding alone cannot determine specifically which domain/motif is responsible for binding when they occur multiple times in either interaction partner. Also, such methods do not permit discovery of novel motifs. Methods using binary interaction data alone overcome some of the problems because binary interaction data sets are more readily produced and aggregators of gold-standard consensus data sets already exist [18].

**Figure 1:**
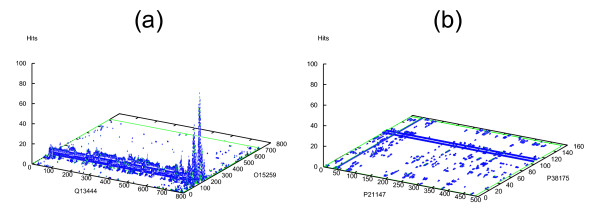
**PIPE landscapes for pairs of yeast proteins**. (a) Interacting pair (O15259 and Q13444); the peak represents the most likely site of interaction on both proteins. (b) Non-interacting pair (YGL055W and YBL090W); low matrix values represent random matches and do not indicate an interaction between the two proteins.

The protein-protein interaction prediction engine (PIPE), previously developed by our group, performs PPI prediction relying solely on query protein sequences and a database of known interacting pairs (binary interaction data). The PIPE method is based on re-occurring polypeptide sequences, which have been found to mediate a large fraction of PPIs [19,20]. By considering every pair of sequence windows from query proteins A and protein B, PIPE generates a three-dimensional landscape which represents the co-occurrence of each window pair among a database of known interacting protein pairs. Peaks in this landscape (Figure 1a) signify a high co-occurrence of the corresponding sequence windows among interacting proteins and suggest an interaction between the two query proteins. A flat landscape (Figure 1b) indicates that each pair of sequence windows only co-occurs infrequently in interacting proteins (as expected to occur by chance) and therefore indicates no interaction. As shown in [19], PIPE has been very successful at making binary predictions of protein-protein interactions with better accuracy than competing methods [21-23].

In this paper, we describe *PIPE-Sites*, a new method for analyzing peaks in the landscapes of co-occurrence reported by PIPE in order to make protein specific binding site predictions. Previous anecdotal evidence from simple "visual inspection" of PIPE landscapes for a small set of examples [24] indicated some correspondence between peaks and known binding sites. This work explores the potential for site prediction at a large (proteome) scale. We present *PIPE-Sites*, an automated way of analyzing landscapes of co-occurrence reported by PIPE in order to make protein specific binding site predictions. To verify PIPE-Sites' predictions, we make use of an experimentally confirmed validation set of protein pairs and the mediating sites on each protein. We evaluate our predictions quantitatively by measuring the distance between the predicted binding site ranges and the experimentally confirmed ranges. Finally, we apply our method to two data sets of novel protein interactions with unknown sites and discuss the results. PIPE-Sites appears to be highly accurate with respect to the experimentally validated data set, and is the first such method operating at proteome scale from binary interaction data alone. In addition, PIPE-Sites shows improved performance compared to existing domain-domain and domain-motif based protein binding site prediction methods such as Riley's domain pair exclusion analysis (DPEA) [12] and DOMINE [25]. PIPE-Sites is available online at http://pipe-sites.cgmlab.org/ where users can enter two query proteins and, if the proteins are predicted to interact, receive a report of the top three landscape peaks and corresponding putative binding sites on each protein.

## Methods

### Training and validation data sets

The PIPE method of protein interaction prediction uses a database of known interactions as training data. These known interactions are given simply as pairs of proteins; no additional annotation is required. We carried out experiments for two species: *Homo sapiens *(human) and *Saccharomyces cerevisiae *(yeast). The known interactions databases were taken from BioGRID [18] and contain 41,678 and 39,899 pairs for human and yeast, respectively.

To validate our site predictions, we used the DOMINO PPI site database [26], which provides specific amino acid ranges for the binding site on each interacting protein. DOMINO extracts results from peer-reviewed articles, representing a wide range of experimental methods. Many records originate from deletion experiments, which have a high rate of false positives due to possible conformational change as outlined above. In some partial records, DOMINO reports a binding site for one protein, but none for the other. These instances and other degenerate cases were excluded from our validation set.

Where DOMINO has binding site data for both proteins, one or both binding sites may refer to an extended region of the protein and hence the size of the binding site is over-estimated. For example, in 5% of records for yeast proteins, the binding site for at least one protein was longer than 100 amino acids (whereas the average yeast protein is 452 amino acids [27]). In an additional 53% of records, phage display was used to screen short peptide arrays for binding against a domain, but the authors did not attempt to find the minimal binding region and instead report the entire domain as responsible for interaction. It is likely that not all amino acids directly participate in binding and thus the true binding site is a subset of the given ranges. Our methodology was adjusted to account for these properties of the validation set (detailed below in "Distance measure"). Despite these shortcomings, DOMINO remains a comprehensive database of binding site ranges, and, after filtering, provided 265 ranges in yeast and 423 ranges in human protein pairs as our validation set (complete lists are given in Additional Files 1 and 2). Note that while 212 protein pairs in human and 67 in yeast from those datasets were also present in our set of known interactions, our training data does not contain any information on binding sites and does not skew our predictive performance. Additionally, while some protein re-occur in the validation set, this is discussed in detail in a later section (Balance In Validation Data), and is shown not to have a significant effect on our results.

Most existing interaction prediction methods operate at the domain-domain or domain-motif level [10-13,28]; some databases collect domain-domain data from several experimental and computational sources [25]. While to our knowledge there is no method which directly predicts binding site amino acid ranges, domain-domain methods can be applied to binding site prediction. To achieve this, we consider every pairwise combination of domains from each query protein and extract amino acid ranges for one or several highest ranking pairs. We applied this technique to compare our method against two existing domain-domain interaction predictors: Riley's domain pair exclusion analysis (DPEA) [12] and the DOMINE domain-domain interaction database [25]. DPEA uses known interacting protein pairs from several organisms to infer domain-domain interactions and provides a log odds score (E-value) for each pair. The published 3005 most statistically significant pairs were used as this method's predictions. DOMINE combines domain-domain interaction data from two methods based on observed physical association in experimental PDB structures and 13 computational methods, including DPEA. For each domain pair, the database specifies which methods confirm the interaction. We produced a simple consensus score by counting the number of methods confirming any given pair, to compare against our method.

Both DPEA and DOMINE identify domains using their accession numbers in the Pfam protein family database [29]. We applied Pfam to our validation set of 265 yeast and 423 human interactions. When comparing against DPEA, we used the same version of Pfam (14.0) because DPEA also makes predictions on a subset of Pfam (Pfam-B) for which accession numbers are not stable across versions. However, since the DOMINE database restricts itself to the stable subset of Pfam (Pfam-A), the latest version of Pfam (version 24.0 released October 2009) was used in order to benefit from the most up-to-date domain annotations available.

Lastly, we applied our method on data sets of interacting proteins for which no protein-specific binding site data is currently available, to demonstrate the potential for detection of binding sites and discovery of novel binding site motifs. First, we obtained from the University of Leuven (personal correspondence) a set of proteins associated with congenital heart defects (CHD) in *H. sapiens*. After screening for interaction every pair of proteins, we applied PIPE-Sites to the 2347 pairs predicted to interact. We also applied PIPE-Sites to 14,438 high-confidence interactions in *S. cerevisiae *previously reported by PIPE as a result of a proteome-wide all-to-all screen [30]. Section "Binding sites in novel human and yeast interactions" summarizes our results on these data sets.

### Distance measure for validation and comparison of binding site predictors

**Figure 2:**
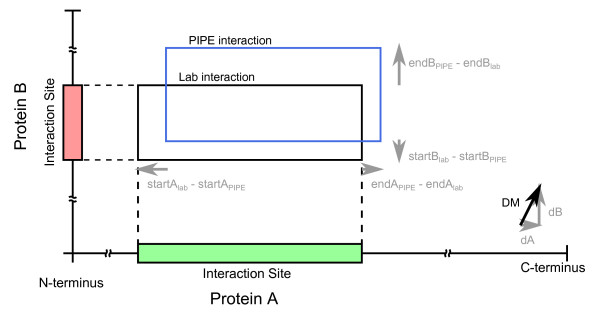
**Distance measure (DM) calculation**. Along each protein, the distance is the maximum difference between start and end positions (*startA_lab _- startA_PS_, endA_lab _- endA_PS_*). When the predicted site is contained within the lab site, the differences are both negative and we clamp the maximum difference at 0 (perfect match). Distances along each protein are scaled and combined vectorially to yield the DM.

The binding sites on two interacting proteins can be represented as a rectangle on an overhead view of the two proteins (Figure 2). These rectangles represent either the sites predicted by our method, or the true sites determined by experiments. In order to objectively gauge the performance of our method, we developed a quantitative method of comparing two rectangles (pairs of interacting regions) for a given interaction Existing prediction methods focus on participating residues on only one side of the interaction, or make predictions at the domain level. In image segmentation evaluation, quantitative comparison of two regions is a well-known problem, and various distance measures have been studied in that context [31]. However, these methods are focused on non-rectangular regions and adopt set-theoretical approaches which are difficult to interpret in a biological context.

We developed a distance measure (DM) between predicted and actual (lab confirmed) protein interaction sites which accounts for overlap of sites on both proteins and combines them into a single value between 0 and 1, where 0 indicates a perfect match and 1 indicates that the rectangles cover opposite ends of the proteins with no overlap (Figure 2). For two interacting proteins A and B of length *proteinA_length _*and *proteinB_length_*, respectively, let *startA_PS_,endA_PS_, startB_PS_, endB_PS _*denote the location of the interaction sites predicted by PIPE-Sites and B let *startA_lab_,endA_lab_*, *startB_lab_*, *endB_lab _*denote the location of the actual (lab confirmed) interaction sites.

We first calculate distances along each protein by subtracting the start positions (*startA_lab _- startA_PS_*) and end positions (*endA_lab _- endA_PS_*) and taking the maximum. When the predicted site is contained within the lab site, the distances are both negative and we clamp the distance at 0 (perfect match) along that protein (Equation 1). This accounts for cases where the experimental data over-estimates the binding site. Then, we combine the distances along each protein into a single overall distance between the two rectangles. The distance along one protein (ΔA) is calculated. The distances for both query proteins are scaled by each protein length and combined by vector addition to yield the final DM (Equation 2). The  factor ensures that the final value is between 0 and 1.(1)(2)

This measure has several useful properties: it penalizes over-prediction of interaction area while rewarding small, specific site predictions, in the presence of very coarse lab data. It weighs boundary errors inversely with protein length, and it is equally sensitive to prediction errors on either protein. Finally, it is numerically comparable for predictions on proteins of arbitrary length.

### PIPE-Sites: Walk algorithm

**Figure 3:**
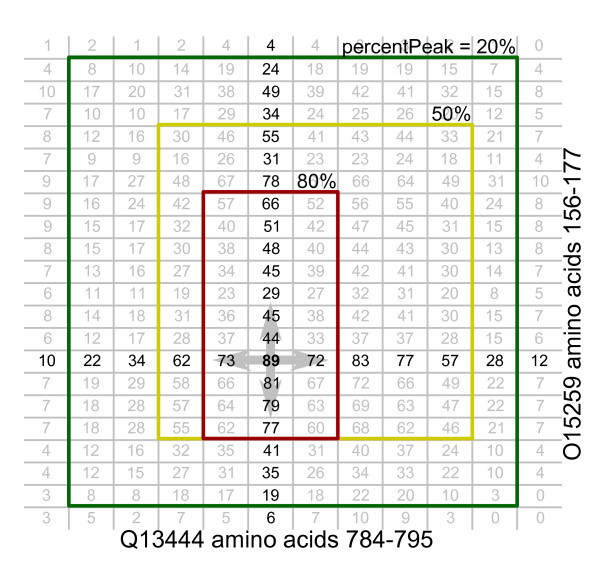
**Walk algorithm**. Starting from a peak, the walk algorithm proceeds in all four axial directions until the landscape height is below a pre-defined percentage of the peak height (percentPeak). As the percentPeak parameter is relaxed, the predicted sites are increasingly larger.

The primary features of PIPE landscapes are peaks and their surrounding hills. Peaks indicate the location of a site of interaction, while the hill leading up to the peak represents the extent of the binding site on each protein. By walking down from the peak until a given height threshold, we can algorithmically determine the cross-sectional area of the peak, and thus, the size of the binding site. The walk proceeds in the four axial directions (up, down, left, right) until the height of the landscape drops below a given threshold (Figure 3). Since the peak height is highly variable, the threshold is expressed as a percentage of the current peak height (percentPeak). Lower values extend the predicted sites by continuing the walk further along the hill surrounding a peak (Figure 3). This allows us to calibrate our algorithm to give more specific site predictions by increasing the percentPeak parameter.

### PIPE-Sites: Multiple peak selection

**Figure 4:**
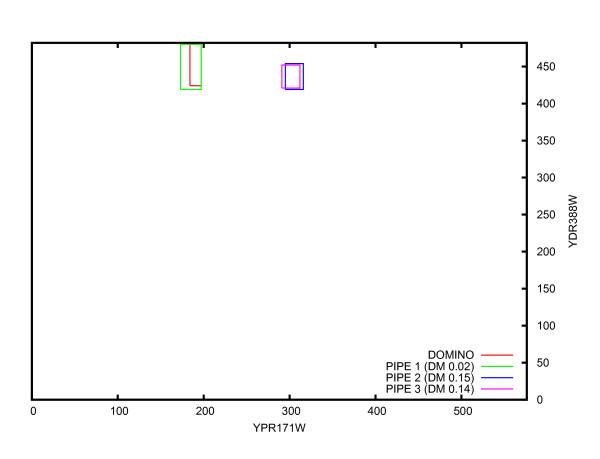
**Overhead view with a lab confirmed range, three PIPE predicted ranges and corresponding DM values**.

Multiple peaks in PIPE landscapes may indicate multiple binding sites (Figure 4). Multiple peaks can also be the result of noisy predictions of interaction due to random sequence window matches, an effect which particularly affects prediction for landscapes with low overall height. In both cases, it is interesting to extract data about more than just the single highest peak in each landscape. This data allows the use of additional features to disambiguate between the peaks or to suggest interesting areas for wet lab experimentation which are likely to yield positive results. To afford the greatest flexibility to the potential users of our method, we generate a ranked list of potential binding sites, ordered by decreasing peak height. Other features can be introduced to improve the selection of a single peak, or multiple experiments can be carried out with higher overall probability of successfully validating the true site(s).

### PIPE-Sites: Handling noisy and spurious peaks

Some PIPE landscapes exhibit very sharp peaks with no supporting hills. These may arise from random matches when searching for similar sequences in the training set and do not indicate a true interaction. In order to exclude these from consideration, we enforce a minimum of one walk step in each direction from the current peak. This excludes spurious peaks without unduly affecting prediction on peaks with significant support.

There are also many pairs for which the PIPE landscape indicates scattered but significant co-occurrence of sequence windows in known interactions. These landscapes are sufficient to predict an interaction but do not contain discrete features for the binding sites. In order to exclude these landscapes from consideration, we require that the tallest peak in the landscape satisfy a given minimum threshold. We experimented with values 5, 10 and 15. The value 10 provided a good compromise between allowing too many noisy peaks and not having tall enough peaks to make any predictions, and was used for the remaining experiments.

### Identification of known domains

The InterPro database [32] integrates protein annotations from several large databases, including PANTHER, Pfam, SMART, ProSite and Superfamily. The InterProScan tool [33] is used to identify domains and motifs in an amino acid sequence without *a priori *knowledge of the protein the sequence was from. InterProScan is available for download for use on a large scale. Functional annotation of predicted binding sites via InterProScan provides biological significance and context to the predictions made by PIPE-Sites. Using the InterProScan tool, the predicted binding site sequences were provided as input, and domains contained within the sequence were the output. We used the latest version of the InterPro database as of this writing (release 29.0).

Domain identification within interactions is significant for two reasons. Identifying known domains confirms that the peak selection algorithm and PIPE method is, in fact, identifying potential binding sites, and not just low-complexity regions within a protein's primary structure. Secondly, the re-occurrence of domains from both participants in the PPI can characterize what types of interactions the method can accurately predict. Additionally, by carefully excluding sequence windows with existing annotations, we are able to discover novel binding motifs that re-occur frequently among our known interactions. Furthermore, if a sequence window does not have any known annotations in InterPro, this means that the sequence is not recognized by any of the constituent databases of InterPro (PANTHER, Pfam, SMART, ProSite, and others) and provides strong evidence that the motif is novel.

## Results and Discussion

### Prediction of binding sites in *H. sapiens *and *S. cerevisiae*

Our validation set, a filtered subset of DOMINO, contains 423 human and 265 yeast interactions. For each human and yeast protein pair, we ran the PIPE algorithm to generate PIPE landscapes and retained only those with PIPE peaks greater than 10 hits (363 for human and 176 for yeast). We then applied PIPE-Sites to predict binding sites for each of these landscapes and selected the top three sites. For each predicted site (3 per protein pair), we calculated the DM with respect to the DOMINO (i.e. lab-confirmed) site for that protein pair. To provide a baseline performance metric, we also generated random predictions by randomly choosing a start location and an end location in the remaining length of the protein and calculated the DMs for three random sites per pair. We analyzed the resulting set of DM values by calculating the enrichment (compared to random interval selection) for small DM values (e.g. 0% ≤ DM < 10%) and negative enrichment for large DM values (e.g. 50% ≤ DM).

**Figure 5:**
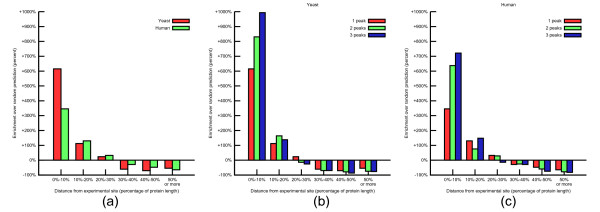
**Enrichment over random site predictions**. Using highest peak only, for yeast and human (a), best of one, two, three peaks peaks in yeast (b) and human (c). For both species, we predict significantly more sites close to the experimentally-confirmed site than random. Yeast performs slightly better due to the much greater coverage of the species interactome by the PIPE known interactions database yeast (36,000 known pairs out of estimated ~50,000 [30]) compared to human (40,000 known pairs but much larger interactome).

We first analyzed DMs for the single highest peak for each pair and compared it to the first random site (Figure 5a). For example, the number of predicted yeast binding sites which are within 10% of the DOMINO site (accurate prediction) is 6 times greater than what would be expected by chance. Conversely, the number of predicted site more than 50% away from the DOMINO site (i.e. inaccurate prediction) is approximately 80% less than what would be expected by chance. While performance is similar between the two species, and both perform significantly better than random (P < 10^-21 ^for human, P < 10^-20 ^for yeast, Kolmogorov-Smirnov test), yeast does perform slightly better than human. The sizes of our PIPE known interactions databases are comparable for human and yeast (41,678 and 39,899 protein pairs, respectively). However, it has been suggested that the human interactome is much larger than for yeast [34], which would explain our improved performance on yeast in terms of relative coverage of total interactions. DOMINE, on the other hand, may benefit from the larger number of known domains annotated per human protein (3.4 domains per protein for the average protein in our validation set) compared to the same value for yeast proteins (2.1 domains per protein on average), resulting in slightly improved performance on human protein pairs. The remaining method, DPEA, is trained on 26,032 pairs spanning 68 organisms, resulting in much weaker single-organism coverage and suggesting a possible justification for its lower performance in general.

We also considered performance on multiple peaks by comparing distributions of the single highest peak, minimum DM of the two highest peaks, and minimum DM of the three highest peaks (Figure 5b, 6c). This represents an experiment where wet lab validation would be attempted on the predicted interaction site corresponding to the highest single peak, the highest two peaks and the highest three peaks. The overall shift of the distribution toward lower DM as the number of peaks increases indicates a general trend for individual DM values to decrease. In particular, the bin "0% ≤ DM < 10%" always increases because with each additional peak considered, sites where the second or third peaks outperform the first peak are assigned to that bin. The average distance between the predicted and lab confirmed site decreases with each additional peak, and is in all cases significantly lower than for the random predictions (Table 1).

**Figure 6:**
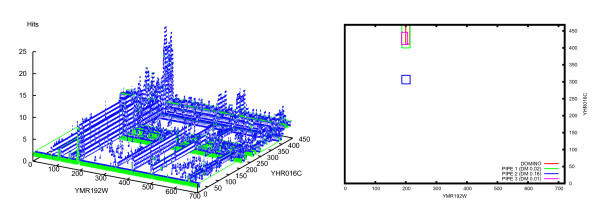
**PIPE predicted sites between YHR016C and YMR192W (*S. cerevisiae*)**.

**Table 1:** Binding site prediction errors (average DM) for human pairs, yeast pairs, and random predictions

Peaks	Human	Yeast	Random
1	0.246	0.218	0.402
2	0.213	0.168	0.320
3	0.188	0.151	0.269

Error is expressed as average distance between the predicted site and lab-confirmed site, averaged over all pairs. Random predictions were made for both human and yeast protein pairs, and the corresponding error is average DM over all pairs.

Finally, we applied a similar methodology on predictions made by the DPEA and DOMINE domain-oriented methods and report average DM for their predictions relative to DOMINO experimental binding sites. We first obtained domain annotations for all proteins in our validation set from Pfam, using specific versions depending on the method, as previously described in Section "Training and validation data sets". Then, for each pair, we retrieved the domain pair predicted most likely to interact according to each method. The amino acid ranges of the domains on each query protein were taken as the predicted binding sites and compared, using DM, to the DOMINO data. We present the average DM for each method applied to each species. Note that as a result of Pfam lookup and the limited number of DPEA strong predictions, some protein pairs had no matching domain-domain interaction. These pairs were simply excluded and average DM was calculated only on those protein pairs for which the method could make a prediction. As DPEA was only able to make predictions for two human protein pairs, we do not consider the sample sufficiently large and do not report results for that experiment. This demonstrates the shortcomings of relying on domain annotations for binding site prediction, as compared to PIPE-Sites, which was able to make predictions on nearly all pairs in the validation set, sacrificing some pairs only to increase the average accuracy of the remaining predictions. See Table 2.

**Table 2:** Binding site prediction error for two domain-oriented methods and PIPE-Sites (this work)

Species	Pairs originally in validation set	Method	Pairs with available predictions	Error (average DM)
Yeast	265	DPEA	32	0.718
Yeast	265	DOMINE	144	0.363
Yeast	265	PIPE-Sites (this work)	174	0.218
Human	423	DPEA	2	N/A *
Human	423	DOMINE	266	0.306
Human	423	PIPE-Sites (this work)	363	0.246

DPEA [12] is a standalone statistical inference method, while DOMINE [25] is a database combining several experimental and computational domain-domain interaction sources. We used a consensus approach to combine the 13 constituent data sources of DOMINE to arrive at a single binding site prediction. Error is expressed as average distance between the predicted site and lab-confirmed site, averaged over all pairs. * Due to the small sample size (only 2 available predictions), it was impossible to calculate a statistically significant average DM for DPEA predictions.

PIPE-Sites itself is not very computationally intensive. We benchmarked the performance of the PIPE-Sites algorithm on modern Intel Core i7 hardware. The average time required for site analysis of pairs in our validation set was 239 ms per yeast pair and 340 ms per human pair (human landscapes are on average larger than yeast landscapes). This is less than half the time required to compute the landscape itself (526 ms on average per yeast pair and 1704 ms per human pair); no attempt has been made to optimize the site analysis code. The theoretical worst case complexity of the site analysis is O(*proteinA_length _× proteinB_length_*), which is due to the search for the location of the maximum value in the landscape. This operation requires a single pass through the landscape and in practice is quite fast.

### Balance in validation data

There are proteins participating in multiple interactions in the dataset of lab-confirmed binding sites. Of the 423 confirmed binding sites in the human dataset, there are 364 unique participating proteins, with one protein involved in 58 PPIs. The yeast dataset contains 265 PPIs with 140 unique participating proteins, and the single most frequently occurring protein is involved in 72 PPIs. Duplication of participants in PPIs could potentially skew the DM scores favourably in the case of an easy prediction and unfavourably in the case of a consistently poorly predicted protein. A consistently poor prediction can arise from a variety of sources, including poor performance of the method on a significant number of landscapes involving the mispredicted protein, a mistake in the lab, the reuse of a single binding site in DOMINO records where multiple in fact exist, or a binding site that changes in the different PPIs a protein is involved in. In order to determine if there was a group of proteins skewing the DM scores in either direction, we measured separately, for each protein pair in the validation set, the contributions from each protein (ΔA and ΔB) to the DM of the highest peak. Then, for each individual protein, we counted its number of occurrences in protein pairs and calculated the average of its individual DM scores.

Tables 3 and 4 show the most frequently occurring proteins in the lab-confirmed dataset: the protein name in column one, the number of occurrences in column two; and the average DM score in column three. The average DM score of the most frequently occurring human protein (P04626 with DM score of 0.233) is close to the overall human average of 0.246. Subsequent entries are also close to the average DM, indicating that these frequently-occurring proteins in general do not skew overall DM results. Some entries, however, Q13444 in particular, show very accurate binding site predictions with a DM of only 0.012. A similar pattern can be seen in yeast proteins, where the most frequently occurring protein has a DM score of 0.149 compared to the average of 0.218. YHR016C is particularly well predicted with a DM score of 0.069. We later discuss the domain associated with the predicted and lab-confirmed binding site of this protein.

**Table 3:** Most frequently occurring human proteins and their average DM scores in the validation data set

Protein	**Freq**.	Error (avg. DM score)
P04626	58	0.233
P00533	48	0.097
P21860	35	0.240
Q13444	21	0.012
P62993	13	0.022
Q15303	11	0.210
O95400	10	0.447

**Table 4:** Most frequently occurring yeast proteins and their average DM scores in the validation data set

Protein	**Freq**.	Error (avg. DM score)
YFR024C-A	72	0.149
YHR016C	48	0.069
YDR388W	33	0.119
YCR088W	25	0.128
YMR109W	24	0.040
YER118C	17	0.150
YBL007C	13	0.198
YBL085W	13	0.138

### Binding site domains

While predicting the binding site subsequence is itself an important problem, relating the subsequence to existing protein annotations is also of great interest to the biology community. Domains provide context for the potential functional uses of a peptide sequence. Identifying domain pairs which were previously not known to interact, or ones that are known to mediate interaction, confirms our hypothesis that our method accurately finds the binding site between two proteins. The co-occurrence of certain domains can explain why two proteins are known to interact. Conversely, predicted sites which are not mapped to existing domains represent potentially novel, undiscovered interaction-mediating domains or motifs.

We used the InterPro database to annotate all human and yeast proteins in the DOMINO dataset and extracted the domain on each protein which most overlaps with the protein's predicted binding site. In order to strengthen conclusions drawn from interacting domain pair analysis, we chose to examine here only those sites for which the prediction's DM with respect to DOMINO data is less than 0.20. These represent predictions where the binding site has been found both experimentally and by PIPE-Sites, and are considered high confidence binding sites. In Tables 5 and 6, we present the top ranked co-occurring interacting domains in high confidence binding sites. For each domain pair, we additionally report whether it is present in DOMINE, a combined database of domain-domain interactions [25]. We later report similar results on a dataset of novel interactions with no experimental binding site information.

**Table 5:** Top ranking co-occurring domains in high confidence binding sites from human

Domain Name A	Domain Name B	DOMINE
SH2 motif	Serine-threonine/tyrosine-protein kinase	Known
Serine-threonine/tyrosine-protein kinase	Insulin receptor substrate-1, PTB	Known
PDZ/DHR/GLGF	Cyclic AMP-dependent chloride channel	Novel
Serine-threonine/tyrosine-protein kinase	Phosphotyrosine interaction domain	Known
Serine-threonine/tyrosine-protein kinase	Serine-threonine/tyrosine-protein kinase	Known
PDZ/DHR/GLGF	Tyrosine-protein kinase, CSF-1/PDGF receptor	Novel
Src homology-3 domain	Serine/threonine-protein kinase-like domain	Known
SH2 motif	SH2 motif	Known
Src homology-3 domain	T-cell adhesion molecule CD2	Novel
7TM GPCR, rhodopsin-like	PDZ/DHR/GLGF	Known

Binding sites included in this analysis had DM ≤ 0.20. Among interacting domain pairs in all sites, 22 out of 47 (46.8%) pairs are reported in the DOMINE database.

**Table 6:** Top ranking co-occurring domains in high confidence binding sites from yeast.

Domain Name A	Domain Name B	DOMINE
Src homology-3 domain	Src homology-3 domain	Known
Src homology-3 domain	Uncharacterised conserved protein UCP037464, actin patch protein 1	Novel
Src homology-3 domain	Adenylate cyclase-associated CAP, N-terminal	Known
Src homology-3 domain	EF-Hand 1, calcium-binding site	Novel
Src homology-3 domain	Protein of unknown function DUF3210	Known
Src homology-3 domain	BTB/POZ fold	Novel
Src homology-3 domain	Septin	Known
Src homology-3 domain	C2 calcium/lipid-binding domain, CaLB	Novel
Src homology-3 domain	Serine/threonine-protein kinase-like domain	Known
Homeobox	Transcription factor, MADS-box	Known

Binding sites included in this analysis had DM ≤ 0.20. Among interacting domain pairs in all sites, 10 out of 15 (66.7%) pairs are reported in the DOMINE database

In Table 5, a number of human domains co-occur, including pairs involving PDZ, a common domain known to mediate protein-protein interactions [35]. Yeast co-occurring domains are highly enriched in Src homology-3 domain (SH3), which is also a ubiquitous domain involved in protein-protein interactions [36]. In particular, SH3 dimerization has been previously shown experimentally [37]. For both human and yeast, approximately half of domain interactions are previously reported [25]. This reflects the ability of PIPE-Sites to elucidate protein binding based on the domain-domain interaction model. Domain pairs not reported by DOMINE could represent novel domain-domain interactions, and can be used to produce an exhaustive list of domain ligands. Table 6 illustrated the ability of PIPE-Sites to enumerate all putative partners of a given binding domain.

### Binding sites of one protein (YHR016C)

While it remains of interest to identify co-occurring domains at a global scale, there are also insights to be gleaned when confirmed and predicted sites are compared at the individual protein level. A candidate protein was chosen (YHR016C) to demonstrate the practical applications and accuracy of the prediction of binding sites via PIPE-Sites. YHR016C and YCR088W are actin-binding, YLR144C may also have a role in actin cytoskeleton assembly, while YMR192W and YPL249C are GTPase activating proteins. Certain GTPases are known actin cytoskeleton regulators [38]. Table 7 displays DM scores for several protein pairs where YHR016C participates. Also included in the table are the domains within the predicted binding site, obtained from the Saccharomyces Genome Database (SGD) [39], and the predicted sequence ranges. All four PPIs are nearly exact predictions (low DM scores). Figure 4 provides a visual depiction of a DM score of 0.02, where the predicted range largely overlaps the lab-confirmed range.

**Table 7:** Predicted binding sites and associated domains for YHR016C and partners

Protein A	Domain A	Range A	Protein B	Domain B	Range B	DM
YHR016C	SH3	426-467	YCR088W	SH3	499-535	0.02
YHR016C	SH3	407-467	YLR144C	Glycosidases	15-36	0.01
YHR016C	SH3	400-467	YMR192W	N/A	187-214	0.02
YHR016C	SH3	407-467	YPL249C	N/A	280-301	0.02

**Figure 7:**
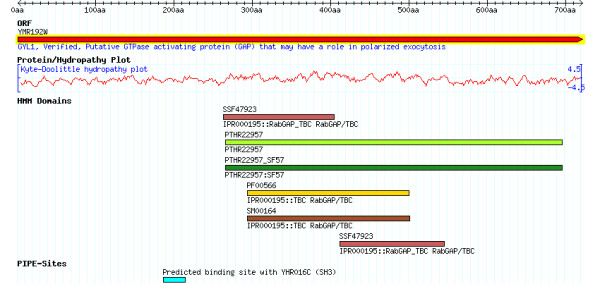
**Saccharomyces Genome Database (SGD) annotation for YMR192W**. Domain annotations are aggregated from several large domain databases (Superfamily, Pfam, SMART, Gene3D, ProSite). The PIPE-Sites predicted binding site with YHR016C's SH3 domain is shown as the last annotation track.

In all the PPIs, approximately the same range is predicted for YHR016C. When compared with the lab-confirmed (411-468, [39]), they are all very near matches. The predicted range contains the SH3 domain, known to be a conserved sequence. It is worth noting that the site predicted in YMR192W (Figure 7) is conserved globally (because of the high PIPE matrix score, see landscape for the pair YHR016C-YMR192W in Figure 6). However, it has not been identified as a recognized domain or motif by ProSite or by other large sequence annotation databases including Pfam [29], SMART [40], or PANTHER [41]. The ability of PIPE to accurately predict confirmed interacting regions not previously known to be domains or motifs is novel and not possible for motif-based binding site predictors.

### Binding sites in novel human and yeast interactions

In this section, we explored the ability of our method to identify novel interaction sites in two datasets of predicted PPIs of interest (one human, one yeast), introduced in Section "Training and validation data sets." In collaboration with the University of Leuven, a list of 2347 human PPIs of interest in congenital heart defects (CHD) was obtained. A dataset of 14,438 novel interacting yeast proteins was obtained from a previous all-to-all screen [30]. As with the DOMINO dataset, we removed landscapes with peaks less than 10. Of the 2347 CHD interactions, 1927 had peaks above 10, while 5216 of 14,438 PPIs remained from the novel yeast dataset. For each site in each pair, we queried the InterPro database for annotations on the interacting proteins to find sites where the binding ranges do not overlap with any known annotations on either interacting protein (Table 8). For 2347 human protein pairs, in which we examined 3 sites per pair, we found 180 sites which have no annotations and could represent novel binding motifs. Among 5216 human protein pairs, we found 958 unannotated sites.

**Table 8:** Summary of results on novel protein datasets.

Species	Total Pairs	Pairs with Well-defined Peaks	Uncharacterized Binding Sites
*H. sapiens*	2347	1927	180
*S. cerevisiae*	14,438	5216	958

The last column indicates the number of sites for which there is no known motif annotation in InterPro and which could potentially represent novel motifs

We note that, at present a binding site was only be predicted for 36% of the novel yeast PPIs. As more PPIs are added to the PIPE database, more re-occurring sequences will be added. In turn, the percentage of PPIs having a peak higher than 10 will increase, allowing more sites to be predicted. While it appears at first glance that predicting human interactions is done with greater coverage than yeast, it is worth considering that the novel yeast proteins were obtained from a proteome wide sample, whereas the human dataset was a specific, targeted family of proteins, which were expected to be more highly enriched in true PPIs since they are functionally related.

### Domain re-occurrence in novel interactions

We now apply the same domain co-occurrence analysis which we previously applied to the datasets of interactions with known binding sites (Section "Binding site domains"). As before, we report whether the domain pairs are present in the DOMINE domain-domain interaction database. In the absence of known binding sites, this provides some validation for the detected interactions and allows us to compare the coverage of known domain-domain interactions between the dataset with known binding sites (where we limited our analysis to accurate predictions) and the dataset of novel interactions. Tables 9 and 10 list the top ranking co-occurring domains in the novel human and yeast protein pairs, respectively.

**Table 9:** Top ranking co-occurring domains in novel human (CHD) interactions.

Domain Name A	Domain Name B	DOMINE	Number of sites
Zinc finger, LIM-type	Zinc finger, LIM-type	Known	28
Zinc finger, LIM-type	Zinc finger, C2H2-type	Known	25
Zinc finger, LIM-type	Actin-like	Known	25
Histone deacetylase	Zinc finger, C2H2-type	Known	23
Serine-threonine/tyrosine-protein kinase	Zinc finger, LIM-type	Known	23
Ankyrin repeat	EGF-like region, conserved site	Novel	22
EGF-like, type 3	EGF-like calcium-binding, conserved site	Novel	20
Zinc finger, LIM-type	Homeobox, region	Novel	16
Helix-loop-helix DNA-binding domain	Zinc finger, LIM-type	Known	15
Zinc finger, C2H2-type	Zinc finger, C2H2-type	Known	15

Out of the 100 most frequently occurring domain pairs, 55% are reported in the DOMINE database

**Table 10:** Top ranking co-occurring domains in novel yeast interactions.

Domain Name A	Domain Name B	DOMINE	Number of sites
Actin-like	Retrotransposon Ty1 A, N-terminal	Known	360
Serine/threonine-protein kinase-like domain	Serine/threonine-protein kinase-like domain	Known	221
Protein kinase-like domain	Serine/threonine-protein kinase-like domain	Novel	170
Sugar/inositol transporter	Protein kinase-like domain	Novel	106
Ras GTPase	Serine/threonine-protein kinase-like domain	Known	99
Ras GTPase	Ras GTPase	Known	95
Ras GTPase	Small GTPase, Rho type	Known	57
ATPase, AAA-type, core	ATPase, AAA-type, core	Known	56
ATPase, AAA-type, core	ATPase, AAA-type, conserved site	Novel	52
Sugar/inositol transporter	Serine/threonine-protein kinase-like domain	Known	51

Out of the 100 most frequently occurring domain pairs, 55% are reported in the DOMINE database.

The occurrence of Zinc finger domains in the dataset of CHD interactions is not surprising as Zing finger domains are associated with DNA-binding, and most of the proteins in the CHD dataset are involved in transcription. In general, the fraction of domain pairs which are present in the DOMINE database is remarkably consistent between the dataset of known binding sites with accurate predictions and the novel interactions, as well as between species. This suggests that the prediction performance of PIPE-Sites is reproducible and consistent on unseen data.

### Potential of finding novel re-occurring motifs

Following peak identification (using 3 peaks) in PIPE-Sites, binding site subsequences were cross-referenced in InterPro for similarity to existing domains and motifs. The number of binding site subsequences not containing an identified domain or motif in InterPro (and therefore not included in Pfam, ProSite, SMART or any other InterPro member database), totalled 180 and 958 for human and yeast, respectively. Table 11 displays some of the highest peaks (most re-occurring sequences) not contained in InterPro from both the human and yeast datasets. These could potentially represent functionally significant binding motifs and merit further investigation.

**Table 11:** Examples of previously unannotated PPI-mediating sequences discovered using PIPE-Sites

Species	Peak Height	Sequence
*H. sapiens*	504	GQVTPPTPPQTAQPPLPGPPPAAVE
*H. sapiens*	234	AAIEPQPSPPHSEPPSVEQPPKPK
*H. sapiens*	195	PIWLQPSPPPQSSPPPQPHP
*S. cerevisiae*	427	ILDGDEDEPEEEDENEGDDEEDTYDS
*S. cerevisiae*	285	DADGDDQTEEGEVEKEQKEEDEEEGPK
*S. cerevisiae*	266	AFDNDESDAQDDANNEKEDDGEEF
*S. cerevisiae*	215	ADQDVEGEDEGGDAIENEDEDEDPSPS

## Conclusions

We have presented and evaluated a novel method for protein binding sites prediction. Our method relies on query protein sequences and a database of known binary interactions only. These data are significantly more abundant and easier to obtain than data such as 3D structures. By first comparing our predictions against a database of lab-confirmed binding sites, we have shown that PIPE-Sites makes accurate predictions which coincide with known binding domain pairs. We also compared our results to two domain-oriented methods, including a comprehensive database used as a consensus method, and demonstrated more accurate binding site predictions by PIPE-Sites. Furthermore, upon applying PIPE-Sites to novel datasets of protein-protein interactions in human and yeast, we have shown PIPE-Sites' ability to reveal domain-domain binding relationships and elucidate potentially novel binding motifs which are as of yet not annotated.

## Authors' contributions

AAB developed the software, conducted the original experiments, and helped draft the manuscript. CP conducted further experiments to characterize the performance of the method, developed the web interface, and helped draft the manuscript. AS completed the InterPro analysis on PIPE-Sites predictions. SP assisted with integration of PIPE-Sites with the existing PIPE framework, and provided insights at all stages of the research. YG contributed to the analysis and discussion of identified domains. JG, FD, and AG conceived of the study, guided the development and evaluation of the method, and assisted with the preparation of the manuscript. All authors read and approved the final manuscript.

## Supplementary Material

Additional file 1 **Pairs of yeast proteins with lab-confirmed binding sites**. This file contains 265 pairs of interacting yeast proteins, with associated amino acid ranges for the binding sites on each protein, extracted from DOMINO [26], and used as validation data. Each line represents one pair, with the following fields tab-separated: ORF name of protein A, ORF name of protein B, start position of binding site along protein A, end position of binding site along protein A, start position of binding site B, end position of binding site B. Click here for file

Additional file 2 **Pairs of human proteins with lab-confirmed binding sites**. This file contains 423 pairs of interacting human proteins, with associated amino acid ranges for the binding sites on each protein, extracted from DOMINO [26], and used as validation data. Each line represents one pair, with the following fields tab-separated: Uniprot accession of protein A, Uniprot accession of protein B, start position of binding site along protein A, end position of binding site along protein A, start position of binding site B, end position of binding site B. Click here for file
